# Subliminal visual stimulation produces behavioural oscillations in multiple frequencies in a visual integration task

**DOI:** 10.1038/s41598-025-85385-5

**Published:** 2025-01-20

**Authors:** Michelle Johannknecht, Alfons Schnitzler, Joachim Lange

**Affiliations:** Institute of Clinical Neuroscience and Medical Psychology, Medical Faculty and University Hospital Düsseldorf, Heinrich-Heine-University Düsseldorf, 40225 Düsseldorf, Germany

**Keywords:** Neuroscience, Cognitive neuroscience, Perception

## Abstract

We perceive our surrounding as a continuous stream of information. Yet, it is under debate, whether our brain processes the incoming information continuously or rather in a discontinuous way. In recent years, the idea of rhythmic perception has regained popularity, assuming that parieto-occipital alpha oscillations are the neural mechanism defining the rhythmicity of visual perception. Consequently, behavioural response should also fluctuate in the rhythm of alpha oscillations (i.e., at ~ 10 Hz). To test this hypothesis, we employed a visual integration task. Crucially we investigated if a subliminal stimulus preceding the target stimulus modulates behaviour. Our results show that behaviour fluctuates as a function of delay between subliminal and target stimuli. These fluctuations were found in the range of theta, alpha and beta oscillations. Our results further support the idea, that alpha oscillations are a functional rhythm for visual perception, leading to rhythmic fluctuations of perception and behaviour. In addition, other frequencies seem to play a role for temporal perception.

**Supplementary Information:**

The online version contains supplementary material available at 10.1038/s41598-025-85385-5.

## Introduction

In our everyday life, we receive a continuous influx of information from our surrounding. A long-standing question is whether this information is processed continuously by our senses. In recent years, the idea of non-continuous perception has gained new popularity. The idea of non-continuous perception states that our visual system works in a rhythmic or discrete manner^1^. Rhythmic perception assumes that stimulus perception is good or less good in certain phases of an underlying rhythm^2^. Discrete perception assumes the existence of perceptual integration windows. Incoming information will be perceptually integrated or segregated depending on whether the information fall within one common or within separate perceptual windows^3^. Both theories – rhythmic and discrete perception—have in common that in their view perception is not a continuous process but rather fluctuates rhythmically over time. It has long been proposed that neuronal oscillations might form the neural basis for rhythmic perception, a theory that has regained popularity in the last years^4–8^. Especially, due to their dominant presence in the visual system, alpha oscillations (i.e., ~ 8–12 Hz) are assumed to be the underlying neuronal rhythm for creating rhythmic visual perception^2^. Therefore, visual perception should fluctuate in the frequency of the underlying alpha oscillation.

Indeed, there is evidence that behavioural responses fluctuate rhythmically. For example, when a visual test stimulus follows a salient, but irrelevant visual stimulus, detection of a test stimulus fluctuates rhythmically (~ 4 Hz) as a function of the distance between test and irrelevant stimulus^9,10^. Similar rhythmic behaviour has been shown for simple visual tasks^9–12^ and more complex visual stimuli in context of image familiarity^13^. Also, for multisensory tasks, studies found rhythmic behaviour. When participants listened to a task irrelevant auditory stimuli and solved then a visual task, behavioural performance fluctuated at ~ 4–8 Hz^14^. While these studies reported rhythmic fluctuations of behaviour, the reported rhythms were often in the range of 4–8 Hz, i.e. in the theta-range. According to the theory of perceptual cycles, however, perceptual processes should fluctuate at the intrinsic frequency relevant for perception in the respective sensory modality, i.e. in the alpha-range for the visual system (~ 8–12 Hz)^2^. Indeed, several studies assigned the rhythmicity of responses in the theta-range to attentional processes rather than perceptual processes. However, Re et al.^12^ pointed out in a feature based attention task that multiple objects were indeed sampled around 4 Hz, but performance on single object fluctuated around 8 Hz. They concluded that these slower frequencies originate from higher frequencies in the alpha range.

While these salient, supra-threshold stimuli can help to understand how attentional resources fluctuate over time and influence behaviour, behavioural fluctuations due to perceptual processes might be overshadowed and not visible. To overcome this problem, subliminal stimuli might be helpful. Subliminal stimuli are physically present but below a perceptual threshold such that observers do not consciously perceive these stimuli. Subliminal stimuli thus unlikely trigger (covert) attentional processes. Although not consciously perceived, subliminal stimuli produce neural activity in early sensory areas, but not in higher order areas^15,16^. Subliminal stimuli might thus influence perception of subsequent stimuli. Previous studies in the tactile domain used subliminal tactile stimuli and found behavioural fluctuations in the beta-range (13–17 Hz)^17^. For the tactile^15,18–20^ as well as visual domain^21–25^ neural signatures of subliminal stimuli have been investigated. While it is not fully clear if the observed neural signatures are best characterized by short lived burst of activity^21^ or whether they are actually long lasting^23^, there is evidence that these stimuli can affect behaviour in various domains^15,19,21,26–28^. We assume that subliminal stimuli can reset ongoing neuronal oscillations without affecting the observer’s attention. By varying the time between subliminal and target stimulus, we can thus manipulate whether the two target stimuli fall within one or two cycles of the oscillation. By systematically shifting these temporal windows, we expect that the perception of the stimuli will rhythmically alter between one or two, i.e. behaviour will show rhythmic fluctuations. It has to be noted that studies differ in experimental design, the type of subliminal stimuli they are using and the conceptualization of what is a consciously perceived stimulus^27^.

In the present study, we want to investigate putative rhythmic perceptual processes in the visual system. While most studies focused on detection tasks, we employed a visual temporal integration task^29^. To disentangle perceptual from attentional processes, we used subliminal stimuli preceding the test stimuli. Similar to previous studies^9,10,12,30,31^, we expected that behavioural responses fluctuate rhythmically as a function of temporal distance between subliminal and test stimulus. Due to the use of subliminal (instead of supraliminal stimuli)^9,10,12,30,31^, we expected to find fluctuations at the frequency relevant for visual temporal integration, i.e. the alpha rhythm.

## Materials and methods

### Subjects

We recruited 20 participants. We excluded one data set, because of some hardware delays causing the sampling delays to be imprecise. Therefore, 19 data sets were analysed (9 females, mean age 25.4 ± 5.2 years SD). One participant was left-handed. All participants had normal or corrected to normal vision and declared to have no neurological disorder. The study was conducted according to the declaration of Helsinki and approved by the ethics committee of the University clinic Düsseldorf. All subjects gave written informed consent bevor participating in this study and confirmed to understand the purpose and requirements of this study. With the written confirmation participants agreed upon their data being published anonymously. Participants were compensated with 13€/hour.

### Paradigm

In a nutshell, we used a visual temporal integration task, which could be solved only if two stimuli separated by a temporal delay were successfully integrated^32^. Crucially, the task stimuli were preceded by a subliminal stimulus with varying delays between subliminal and task stimuli.

**Fig. 1:**
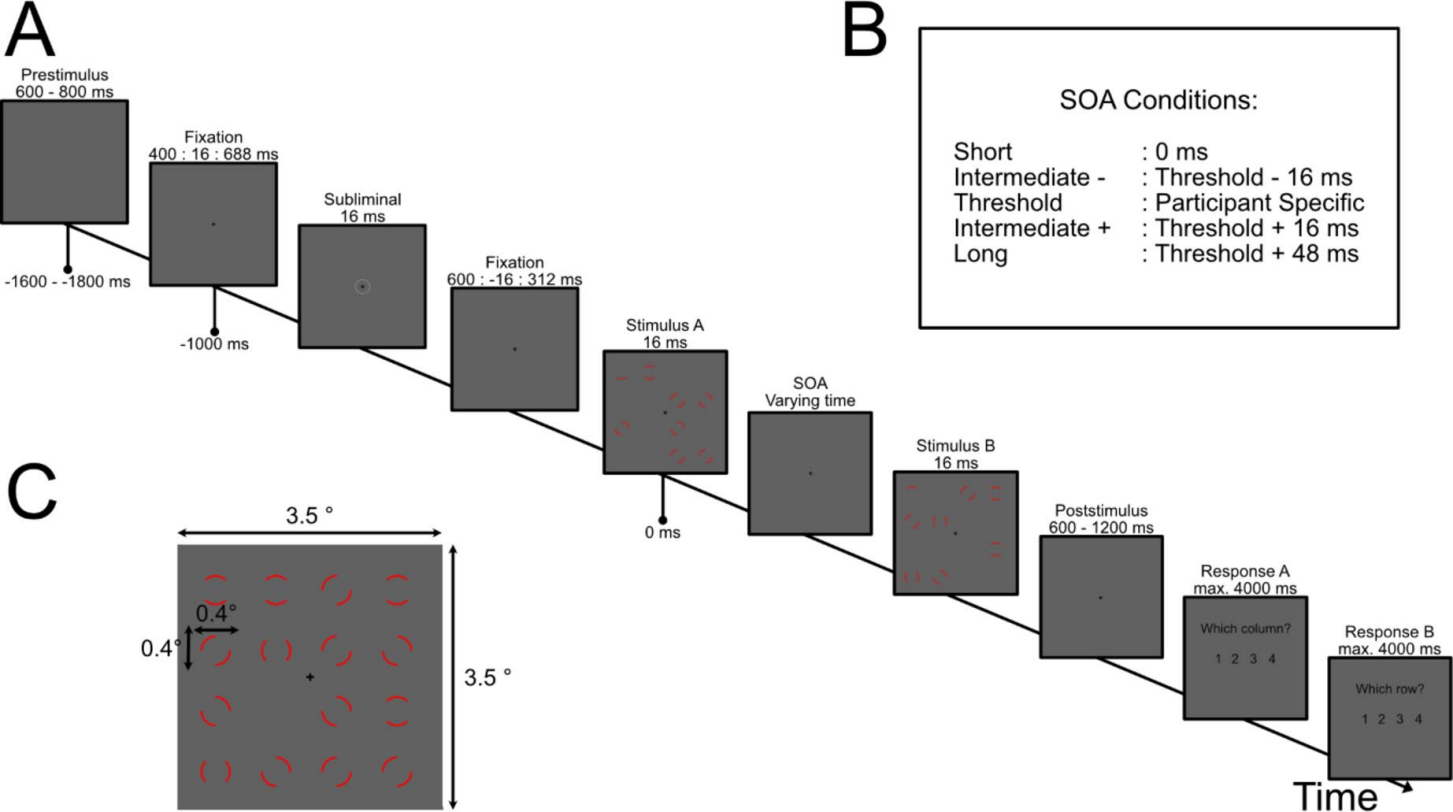
Paradigm. (**A**) The experiment started with a prestimulus period where only a dark grey screen was shown (for 600–800 ms). Afterwards, a fixation cross was presented for 1000 ms. Within these 1000 ms, additionally a subliminal stimulus was shown for 16.6 ms (one frame) at random positions between 312 and 600 ms before the test stimulus. The subliminal stimulus consisted of a ring around the fixation cross (visual angle 0.8°). Its grey value was adjusted to the perception level of the participant. Next, the two test stimuli were shown for 16.6 ms, each with a varying stimulus offset asynchrony (SOA) (see **B** for SOA condition). A poststimulus period followed, where a fixation cross was shown for 600 ms up to 1200 ms. Then the first and second response prompt followed (each could be displayed for up to 4000 ms). (**B**) The different SOA condition used in this experiment. The threshold SOA was individually defined to achieve 50% accuracy. (**C**) An example of a fully integrated test stimulus.

In more detail (Fig. 1A), during a prestimulus period the dark grey screen was shown for a random duration between 600 and 800 ms. Next, a fixation cross was shown for a duration of 1000 ms. Within this period, additionally the subliminal stimulus was shown for 16.6 ms at a random time point between − 600 ms and − 312 ms (in steps of 16.6 ms). After the fixed fixation period of 1000 ms, the two task stimuli were shown. The two task stimuli consisted each of seven full and one-half annuli placed on random positions on a 4 × 4 grid. If both images were superimposed, all grid positions were filled except one empty location (see below for a detailed description). Each image was shown for 16.6 ms and the images were separated by a stimulus offset asynchrony (SOA). We used different SOA condition similar to previous studies^17^. The different SOA conditions consisted of a short SOA of 0 ms (i.e., both images shown in direct succession), the threshold SOA obtained from the staircase (see below for a description), two intermediate SOAs (i.e. threshold SOA ± 16.6 ms; called intermediate plus/minus) and a long SOA condition (threshold SOA ± 48 ms) (Fig. 1B). These conditions were used to control for the behavioural performance of the participant. The threshold SOA was set to be around 50% accuracy. Therefore, we included control conditions to ensure participants were performing the task as expected and were not guessing. In each block, we presented for each subliminal-target-delay 10 trials of the threshold SOA and 1 each of the intermediate plus and minus condition and of the long and short SOA in pseudo-random order. After both target stimuli were presented, the fixation cross was shown for a random duration between 600 and 1200 ms. Afterwards participants had to report the position of the column and the row of the empty location by pressing buttons with the right hand. First, they reported the column by pressing one of four buttons. Next, they reported the row, again by pressing one of four buttons. After the response or if no response was given within 4000 ms, the next trial started.

We additionally included catch trials for the subliminal stimulus, which were randomly interspersed between the other trials. In these catch trials, only the subliminal stimulus was presented, and participants were asked if they had seen the stimulus. They had to respond “yes” or “no”, by pressing one of two buttons on the response button box. The buttons were randomly assigned to the answer choice. We used real and fake catch trials, during real catch trials the actual subliminal stimulus was presented and during fake catch trials the fixation cross on the dark grey background was continuously presented. There was a 10% chance that a trial could be a catch trial. Fake and real catch trials appeared with 50% chance, each.

To increase the number of trials, we split the experiment into two separate sessions, each on separate days. For each session, we repeated the two staircases. Each session included 20 trials of the threshold SOA per sampling delay (380 trials per session) and two trials of the other SOA conditions per sampling condition (38 trials combined for the other SOA condition per session). Each session lasted between 45 and 60 min of recording. The participant did a short training session of 2 to 5 min before starting the experiment.

### Stimuli

The task stimulus consisted of two separate, complementary images. Each image had seven full and one-half red [RGB 1 0 0] annuli placed on random positions of a 4 × 4 grid on a dark grey background [RGB 0.38 0.38 0.38]. In the centre of each image was a black [RGB 0 0 0] fixation cross (Fig. 1C). When both images were superimposed, all positions of the 4 × 4 grid are filled except one empty location. The size of the grid was 8 cm by 8 cm (visual angle of 3.5° by 3.5°). Each annulus was 1 cm by 1 cm, and evenly spaced on the grid (Fig. 1C). Twenty test stimuli (i.e., 40 single images in total) were generated prior to the experiment. The task was adapted from Wutz et al.^29^ and included an integration and segregation task (half annulus). The segregation task was not used in this experiment, but we decided not to change the stimulus.

The subliminal stimulus was a ring around the fixation cross. The ring had a diameter of 1.8 cm (visual angle of 0.8°). The colour value of the circle ranged between 0.38 (no contrast to the background, 0% Weber contrast) to 0.57 (high contrast, 40% Weber contrast). The colour value was adapted individually in a staircase method (see below), so that the stimulus was just not consciously perceived.

The stimuli were presented via a projector (Panasonic, PT-DW700E; 60 Hz refresh rate) and projected onto a translucent screen using a mirror system. The screen was 140 cm in front of the participant. Stimulus presentation was controlled by the software Presentation (Neurobehavioral System, Albany NY, USA).

### Staircase

Prior to the experiment, we ran two separate staircase procedures: In one staircase procedure, we defined the contrast threshold of the subliminal stimulus. In the other staircase procedure, we defined the threshold stimulus onset asynchrony (SOA) for the task stimuli.

In the staircase for the contrast threshold, we presented the participant on each trial only the subliminal stimulus. The starting RGB value was 0.47, so the contrast difference is around 21.2% (Weber contrast). The subliminal stimulus was presented for 16.6 ms and participants had to report via button press if they had seen the stimulus (“yes” or “no”). After each “yes” the grey value was decreased by 0.01 (contrast decreased by 2.6%). When the participant reported ten times in a row “no”, the grey value was increased by 0.01 (contrast increased by 2.6%). If the participant reported 20 times in a row “no”, the staircase terminated, and the momentary grey value was taken as the value for the subliminal stimulus in the subsequent main paradigm. In between trials, stimuli with random grey values were presented to the participant. These randomly chosen stimuli were not evaluated during the staircase. Participants had to respond either with their index finger or with middle finger. We assigned the response choice (yes/no) randomly to these two buttons for each trial.

During the staircase for the SOA of the task stimuli, we presented the participant only the task stimuli. Participants’ task was to find the empty location in the 4 × 4 grid of annuli. This task could be solved only by superimposing both images. Participants reported the position in two steps: First, they had to press one of four buttons on a button box to report the column. In the second step, they had to press the same buttons to report the row. The stimuli were presented for 16.6 ms, each, separated by a SOA. The starting SOA was always set to 26 ms. When the participant reported twice in a row the correct position, the SOA was increased by 16.6 ms. When the participant answered twice in the row incorrectly, the SOA decreased by 16.6 ms. If the SOAs of the last 20 trials were stable (i.e. variance < 0.5), the staircase terminated, and the momentary SOA was taken as the threshold SOA in the subsequent main paradigm. In between trials, random SOAs (between 0 ms and 144 ms, in steps of 16.6 ms) were presented. These randomly chosen SOAs were not evaluated during the staircase.

### Behavioural analysis and statistics

Data were analysed in Matlab (Version R2023b) using the FieldTrip Toolbox (Version 20210825)^33^ or in Python (Version 3.10.12) using Google Collaboratory.

We analysed each participant’s responses as a function of SOA between the two target stimuli, and then averaged the performance for each SOA across participants. To this end, we pooled the response per SOA across all subliminal-target-delays. This analysis was done for each session separately. To test for a systematic difference in accuracy across SOA or across sessions, we ran an ANOVA with post-hoc pairwise comparisons (Tukey Kramer test).

Additionally, we evaluated the performance for the catch trials, by calculating for each session the average “yes” response per participant and then averaged across participants. To test for significant differences, we ran an ANOVA.

Second, for each participant and subliminal-target-delay, we calculated the accuracy for the threshold SOA trials. The data were then linearly detrended and demeaned. The analysis was done separately for each subject and session. Due to the relatively short time window for which we could sample the subliminal-target delay, spectral resolution was relatively poor. To increase the spectral resolution, we zero padded the data to 1 s and we computed a fast Fourier transform on this data. Because we sampled the subliminal-target-delay every 16.6 ms, our frequency resolution was 60 Hz, so the Nyquist frequency was 30 Hz, therefore the FFT was performed between 1 and 29 Hz. We used for each time window a single taper resulting in a spectral smoothing of 3 Hz. This spectral smoothing was done, to compensate for spectral leakage and inter individual differences due to the comparably poor spectral resolution induced by the relatively short time window for the analysis. Afterwards the power spectrum of the behavioural data was averaged across sessions and then across participant. Afterwards the power spectrum of the behavioural data was averaged across sessions and then across participants. As a supplementary analysis we detrended the data with a second order polynomial, as it was done in previous studies^12,34–38^.

To test whether the power spectrum showed significant peaks (i.e., behavioural data fluctuated rhythmically), we compared the spectra to surrogate data. First, we constructed surrogate data by shuffling the accuracy values (raw values) randomly in time and repeated this 1000 times. For each repetition, we detrended and demeaned the data and then calculated an FFT with the same settings as for the observed data. We calculated the median of the surrogate repetitions per subject and then averaged over session per subject^9,17,31^.

Because this method of time shuffling received some critic^39^, we used two additional methods to generate surrogate data. First, we created random accuracy data per sampling delay. Second, we used an autoregression method for generating surrogate data.

For the random accuracy surrogate dataset, we simulated data separately for each participant and session. For each sampling delay, we modelled response values randomly as “correct” or “incorrect”. The number of repetitions at each sampling delay was identical to the number of trials in the observed data. Since observed behavioural data showed that performance was not perfect at 50% accuracy for the threshold SOA, we added an offset to the random accuracy values. This offset was based on the average performance of the respective participant in that session for the threshold SOA. Afterwards, we averaged the resulting random accuracy values across repetitions per sampling delay and repeated this step 1000 times. For each repetition, we calculated an FFT with the same settings as described above and then calculated the median of the surrogate repetions of the power values and averaged over session, for each participant separately. This procedure was inspired by a shuffling procedure by VanRullen^40^, while we did not shuffle between groups but between two possible outcomes. We included this analysis because it is an easy-to-understand procedure and is seemingly less controversial compared to time shuffling. Therefore, we see it as an alternative to time shuffling and want to compare if both methods produce qualitatively similar results.

The AR model was constructed in Python based on Brookshire^39^. We used the “statsmodel” module and constructed an ARIMA process with an autoregression parameter of 1:

1 $$\:{X}_{t}=c+\:{\Phi\:}{X}_{t-1}+\:{\epsilon\:}_{t}$$

where $\:{X}_{t}$ is the modelled response, $\:c$ is a constant, $\:{\Phi\:}$ the autoregression constant, $\:{X}_{t-1}$ the data at time point t minus one and finally, $\:{\epsilon\:}_{t}$ is noise^13,39^. The input for the model was the detrended accuracy data. The noise is based on the standard deviation of the residuals estimated by the model. Based on the estimated parameters from the model for each subject and session an ARMA (Auto Regressive Moving Average) process was modeled, from this, we generated 1000 surrogate data sets with the same length as the original data set. This data was then saved and imported to Matlab, where we calculated for each repetition the FFT, after detrending and demeaning the data. The median of the surrogate repetition was calculated and the averaged over session for each participant.

For all three approaches, we tested the observed data against the surrogate data by means of a cluster-based permutation test^41^. The procedure was identical for all three sets of surrogate data. First, we used a t-test to statistically compare for each frequency the power values of the observed data and the surrogate data. Then, we applied a threshold (t = 1.734, alpha = 0.05) to the t-values and t-values of neighbouring frequencies exceeding this threshold were clustered. The t-values within each cluster were summed and this value served as the cluster statistic of the observed data. Next, we shuffled randomly power values between observed and surrogate data sets. This was done 10,000 times and for each repetition cluster statistics were computed as described above. For each repetition, the largest cluster was selected. Afterwards, we compared the observed cluster with the surrogate cluster distribution and calculated the respective p values.

## Results

Participants performed a visual temporal integration task in which task stimuli were presented with varying SOAs and an empty location on a 4 × 4 grid had to be detected. The task could only be solved, if the two stimuli were perceptually integrated. Crucially, the two task stimuli were preceded by a subliminal stimulus with varying delay before the task stimuli. To increase the number of trials, participants performed the task in two sessions on two separate days.

### Group level performance

**Fig. 2:**
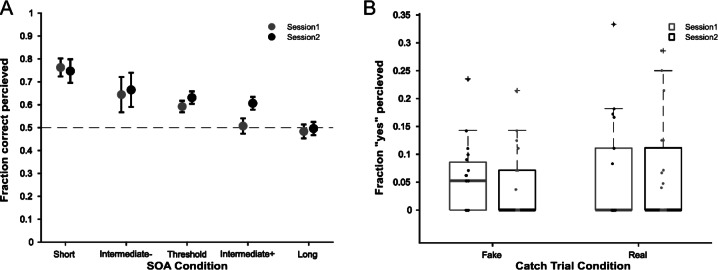
Behavioural data. (**A**) Group level results of the accuracy as a function of SOA. Data are shown individually for each session. The dashed line indicates 50% accuracy. The black lines indicate the standard error of the mean. The results of an ANOVA showed a main effect of SOA condition on the accuracy (F(4,155) = 13.27, *p* < 0.01) but no effect of session (F(1,155) = 1.48, *p* = 0.226), and no interaction effect (F(4,155) = 0.64, *p* = 0.637). (**B**) Results for the catch trials for each condition. Data are shown individually for each session. Fake catch trials were catch trials without a subliminal stimulus presented and real catch trials with a subliminal stimulus presented. The thick line within the boxes indicates the median. The plus signs indicate outliers. The small dots represent individual data points. An ANOVA showed no effect of catch trial condition (F(1,75) = 0.81, *p* = 0.372), session (F(1,75) = 0.04, *p* = 0.84) and no interaction effect (F(1,75) = 0.15, *p* = 0.7).

First, we tested statistically if session and/or SOA between the task stimuli influence the behavioural performance of the participants. The average threshold SOA for session one was 42 ms (± 28.43 ms) and for session two 40.32 ms (± 28.9 ms). We found a statistically significant main effect of SOA condition (F(4,155) = 13.27, *p* < 0.01) but no effect of session (F(1,155) = 1.48, *p* = 0.226) and no interaction effect (F(4,155) = 0.64, *p* = 0.637) (Fig. 2A). A post-hoc multi-comparison test (Tukey Kramer test) revealed a significant difference between the short SOA and threshold SOA (*p* < 0.01), between the short SOA and the intermediate plus SOA (*p* < 0.01), between the short SOA and the long SOA (*p* < 0.01), between the intermediate minus SOA and the long SOA (*p* < 0.01) and finally between the threshold SOA and the long SOA (*p* < 0.01).

### Responses to subliminal stimuli

Next, we analysed if participant did not perceive the subliminal stimulus. We statistically compared participants’ “yes” (i.e. seen) reports in the real and fake catch trials. We found no significant effect of catch trial type (fake vs. real) (F(1,75) = 0.81, *p* = 0.372) or session (F(1,75) = 0.04, *p* = 0.84) and no significant interaction effect (F(1,75) = 0.15, *p* = 0.7) (Fig. 2B).

### Rhythmic behaviour

**Fig. 3:**
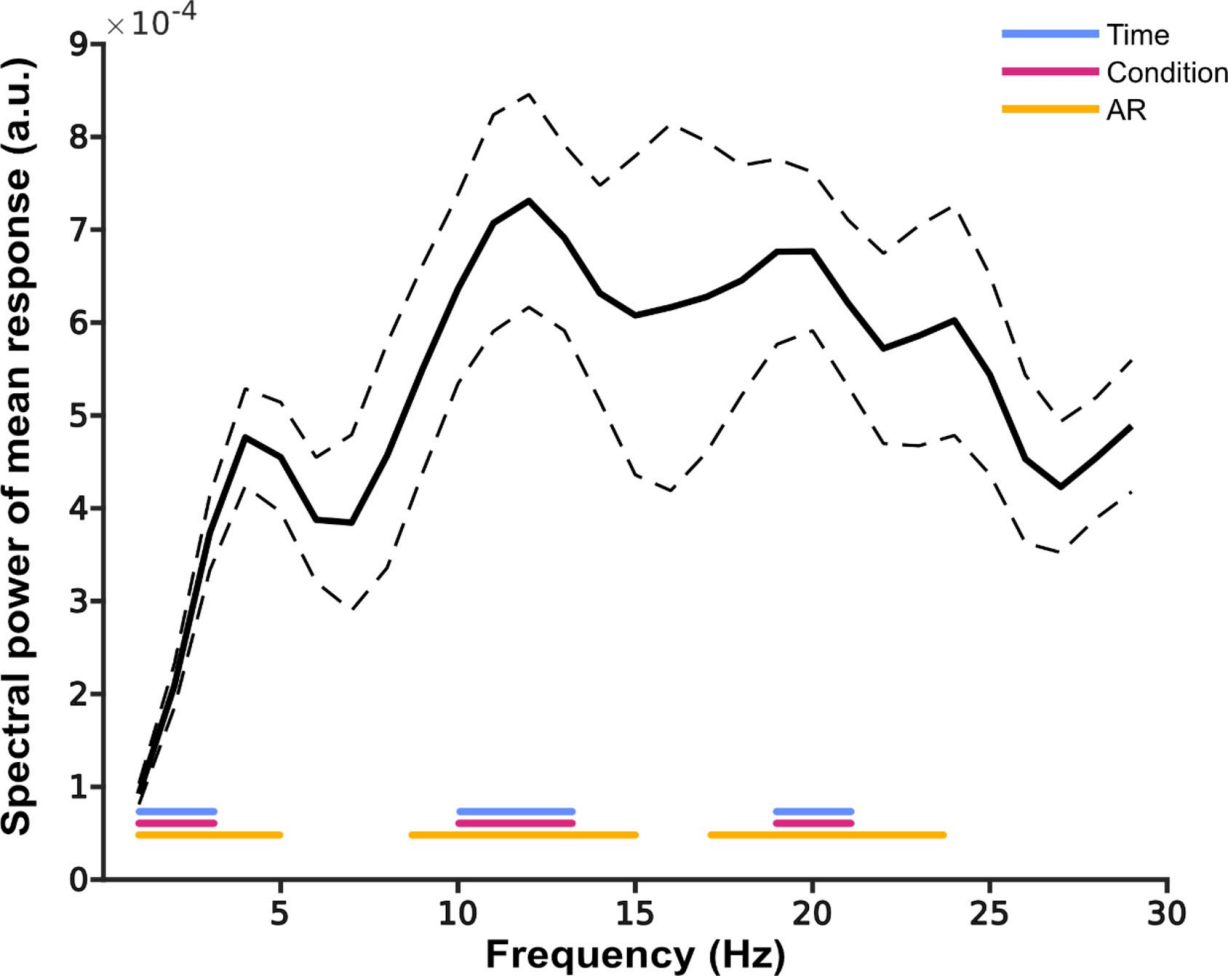
Spectral power of the responses (thick line: mean across participants, dashed line: standard error of the mean). Coloured lines at the bottom indicate frequencies, which are significantly different from the random surrogate data (all *p* < 0.01). The different colours indicate the different methods used to generate surrogate data (see Methods for details).

Finally, we analysed whether participants’ performance fluctuates rhythmically as a function of delay between subliminal stimulus and target stimuli^17^. The results of the cluster-based permutation test revealed for each surrogate data set significant positive clusters. Positive t-values indicate stronger rhythmicity in this frequency compared to the surrogate distribution (Fig. 3), comparison between mean spectral power and surrogate data is shown as a supplementary plot (see supplementary Fig. 1). For the time shuffled data set, we found a significant cluster between 1 and 3 Hz (cluster statistic = 8.3, *p* < 0.01), between 10 and 13 Hz (cluster statistic = 8.4, *p* < 0.01) and between 19 and 21 Hz (cluster statistic = 6.2, *p* < 0.01). For the random accuracy surrogate data, again we found three positive clusters between 1 and 3 Hz (cluster statistic = 9.3, *p* < 0.01), between 10 and 13 Hz (cluster statistic = 8.8, *p* < 0.01) and 19–21 Hz (cluster statistic = 6.4, *p* < 0.01). For the AR shuffling again, we found three positive clusters 1–5 Hz (cluster statistic = 21.82, *p* < 0.01), between 9 and 15 Hz (cluster statistic = 22.24, *p* < 0.01) and between 17 and 24 Hz (cluster statistic = 20.92, *p* < 0.01). Single subject power spectra are shown in supplementary Fig. 2. To control whether the significant clusters in the low frequencies are just a side effect of the short time windows used for the analysis and/or the linear detrending, we reran the analysis with a second order polynomial detrending approach. The additional detrending method led to essentially identical results with all clusters still being significant (*p* < 0.017), except the cluster 1–3 Hz in the time shuffled dataset.

## Discussions

In this study, we analysed the effect of subliminal stimulation on the behavioural response in a visual integration task. We applied three different methods to produce surrogate data, ensuring that the analysis pipeline itself did not artificially bias our results. Independent of analysis pipeline, we found behavioural fluctuation, in a delta/theta frequency range (1–3 Hz), in the alpha range (9–15 Hz) and in the beta range (17–24 Hz). Our initial hypothesis was that the frequency of behavioural oscillations should match the frequency of relevant oscillations in the respective modality. That is, we expected to find behavioural oscillations mainly in the alpha range. While our results confirm this hypothesis, we also find behavioural fluctuations in the theta- and beta-range.

We found behavioural fluctuations in the range of the assumed relevant neural oscillation, similar to the study of Baumgarten et al.^17^. In their study, Baumgarten et al.^17^ investigated tactile discrimination ability. They presented, two suprathreshold tactile stimuli separated by a stimulus offset asynchrony and participants had to discriminate between two or one perceived stimuli. Prior to the tactile discrimination task, a subliminal tactile stimulus was presented at different time delays between subliminal and suprathreshold stimuli. Baumgarten et al. reported behavioural oscillation between 13 and 24 Hz, i.e. in the beta range, supporting their hypothesis that for the somatosensory domain beta oscillations are the functional frequency sampling tactile perception. In line with this hypothesis, we found behavioural oscillations in the range of the functional frequency in the visual modality, i.e. in the alpha range.

Studies using visual tasks reported behavioural oscillations, in the alpha and at higher frequencies^10,13^. Song et al.^10^ used a visual discrimination task preceded by an above threshold cue. They reported behavioural oscillations at a frequency of 2–5 Hz, confirming previous studies^9^. Interestingly, behavioural performance also showed fluctuations in the alpha-/beta range (8–20 Hz). Song et al.^10^ concluded that the slow oscillation samples spatial locations and the alpha oscillation is involved in the attentional sampling process. Liu and Melcher^13^, investigated in a memory task if face familiarity modulates behavioural oscillation in a gender discrimination task, showing either familiar or unfamiliar faces. They found slow oscillations (2.7 Hz) for unfamiliar faces and fast oscillations (12.9 Hz) for familiar faces. They concluded that task demand could influence perceptual sampling. Wutz et al. used the same task as we did in the present study. They found that alpha oscillations were modulated by task demands, i.e., whether participants’ task was to integrate or segregate the stimuli^42^.

In addition to the alpha rhythm, we found behavioural fluctuations at slower frequencies (~ 1–3 Hz). This analysis yielded significant clusters in the low frequencies (~ 1.5 Hz). As a supplementary analysis to control whether the effects found in low frequencies are substantial, we detrended the data with a second order polynomial, as it was done similar to previous study^12,34–38^. Slower oscillations in behavioural performance have been associated with attention mechanisms^12,31,43^, with supra threshold stimuli attracting the attention of the observer. In our study, we used subliminal stimuli to modulate perception. The rationale to use subliminal stimuli was to avoid such covert attentional processes that putatively overshadow potential perceptual processes at alpha rhythms. A crucial question is whether the subliminal stimulus was indeed not consciously perceived. By presenting only subliminal stimuli interspersed between the main paradigm, we aimed to control whether the subliminal stimuli were perceived. Our data show that we can be confident that the subliminal stimulus was indeed below perceptual threshold.

It should be noted that we used short time windows of 288 ms, which do not allow full cycles of oscillations below 3.47 Hz. To control for the fact that these effects in the slow frequencies might be a confound of the short analysis windows, we additionally applied a second order polynomial detrending approach. While the results remained essentially identical with the second order detrending approach, arguing that the effects in the low frequencies might be true patterns of behaviour, we would like to stress caution when interpreting these results. Given that we could not analyse full cycles in these low frequencies, it remains unclear whether we found real oscillatory patterns. Slower oscillations in behavioural performance have been found in other studies and these studies have associated these rhythms with attention mechanisms^12,31,43^, with suprathreshold stimuli attracting the attention of the observer.

While we carefully controlled that the subliminal stimuli were not consciously perceived and do not attract covert attention, it seems that our task still involved some kind of attention. Studies have shown that suprathreshold stimuli that were not consciously perceived could still modulate attention^21,27^ While not being consciously perceived, also subliminal stimuli might involve some kind of (unconscious and overt) attentional processes. These overt attentional processes might be reflected in the slow oscillations of behaviour in our task. Alternatively, it might be that attention fluctuates naturally over time^44^ and we caught these natural fluctuations with our task. Wutz et al.^30^ used the same task as we did in the present study, but participants had to perform either an integration task as in our study or a segregation task. Wutz et al. found an oscillation at the theta-band (3–7 Hz) in the prestimulus period. Whether stimuli were integrated or segregated depended on a phase shift in this oscillation.

Finally, we also found behavioural oscillations in the range of beta oscillations. In a previous study, we used the same task but without presenting a subliminal stimulus prior to the task stimulus. In this study, we measured neural activity with MEG in addition to the behavioural performance. We found that participants’ behaviour (i.e. correct vs. incorrect identification of the empty location) correlated with the phase of neuronal oscillations in the range of alpha oscillations, but the effect also extended to the beta oscillations (8–20 Hz; see also^32^). Other studies found that task difficulty modulated the frequency range. And the frequency range was functionally relevant for behavioural performance. More difficult tasks or higher task demands could shift the frequency relevant for the task to higher frequencies up to beta frequencies^13,30,45^. An alternative explanation could be inter-individual differences between participants. When comparing the individual behavioural spectra, we can see that some participants show only one peak (in the alpha or beta range), others multiple peaks. It could be that individually experienced task difficulty is reflected in different frequencies of behavioural fluctuations.

In sum, all these different factors could explain the differences in the individual spectra resulting in three different peaks on group level. While the behavioural fluctuations in the alpha-range confirm our initial hypothesis, other frequency ranges might also be functionally relevant for temporal visual perception.

With this study, we aimed to delve deeper into the mechanism by which our visual system operates. We investigated putative rhythmic perceptual processes in the visual system. We observed rhythmic fluctuations of the behavioural responses, suggesting a non-continuous process. These results are in line with the results predicted by the model of perceptual cycles. An alternative explanation might be that the phase of the neuronal oscillations determines whether the processing of a stimulus will be facilitated (at a “good” phase) or inhibited (at a “bad” phase”). Also, according to this theory, perception should rhythmically fluctuate with varying delays between subliminal and target stimulus. Without further exploration of the underlying neuronal framework, we cannot differentiate between a rhythmic or discrete process. Further analysis using the additionally recorded MEG data will be necessary to clarify these two theories. Perceptual cycles refer to the hypothesis that cycles of neuronal oscillations might reflect the neural basis of temporal integration windows^2,17,46^. If two stimuli fall within one cycle/window, they will be integrated to one stimulus, but if they fall into two cycles/windows, they will be perceived as two separate stimuli. These perceptual processes of temporal integration should happen at other frequencies (8–12 Hz in the visual domain) as the attentional processes (4–8 Hz). As mentioned above, several studies assigned the rhythmicity of responses in the theta-range to attentional processes rather than perceptual processes. Whether perceptual processes lead to the proposed fluctuations in the alpha range remains unclear.

At this moment, we can only speculate about the mechanism how the subliminal stimulus modulates behavioural responses. Previous studies assumed that supra-threshold stimuli can reset the phase of the ongoing neuronal oscillation and thus lead to fluctuations of behaviour^31,48^. In addition, it has been reported that subliminal stimuli can induce evoked response^18,20,21,23^ and phase resets of neuronal oscillations^48^, which might lead to behavioural fluctuations^17^. In the present study, we additionally recorded neural activity with MEG. An obvious next step in a future study is to analyse these data with respect to putative phase resets of neuronal oscillations induced by the subliminal stimulus and its putative relation to the behavioural fluctuations.

Another limitation of our study was the short analysis window. Future studies could benefit from longer prestimulus periods to investigate the frequency specific signatures of behavioural fluctuations further. This limited our spectral resolution of the behavioural data. Zero-padding and Spectral smoothing was applied to compensate for this lack of resolution and smooth over inter-individual differences. However, further studies would benefit from longer prestimulus periods to elongate the analysis time window.

Additionally, future studies might directly compare the influence of subliminal and suprathreshold stimuli on behaviour and neural activity. Such comparisons might shed new light on the processes underlying covert/overt attentional sampling and perceptual sampling.

## Conclusion

In the present study, we showed that a subliminal stimulus could induce fluctuations of participants’ performance in a visual temporal discrimination task. We found that behavioural performance fluctuated in the alpha rhythm, as well as at slow theta rhythms and comparably high beta rhythms. These results are in line with the hypothesis that our visual system operates non-continuously. The data, however, do not allow us to distinguish whether the underlying process is a rhythmic facilitation or a discrete process. In further studies, we will analyse electrophysiological data to investigate if prestimulus alpha phase modulates temporal visual perception.

## Electronic supplementary material

Below is the link to the electronic supplementary material.

Supplementary Material 1

## Data Availability

Behavioural data will made public, as well as the code the data was analysed with. The scripts for pre-processing are available as well so that interested readers can understand how the data was handled before analysis. The data and code can be found under following link: https://osf.io/j8sxp/?view_only=79f9f15691ac4858886217a24018495a.
