# Supplementary material for: Subliminal visual stimulation produces behavioural oscillations in multiple frequencies in a visual integration task
Source: Sci Rep. 2025 Jan 20;15:2531. doi: 10.1038/s41598-025-85385-5 (PMC11747247; doi:10.1038/s41598-025-85385-5)
Supplement: Supplementary file 1 — Supplementary Material 1 [file 41598_2025_85385_MOESM1_ESM.pdf]

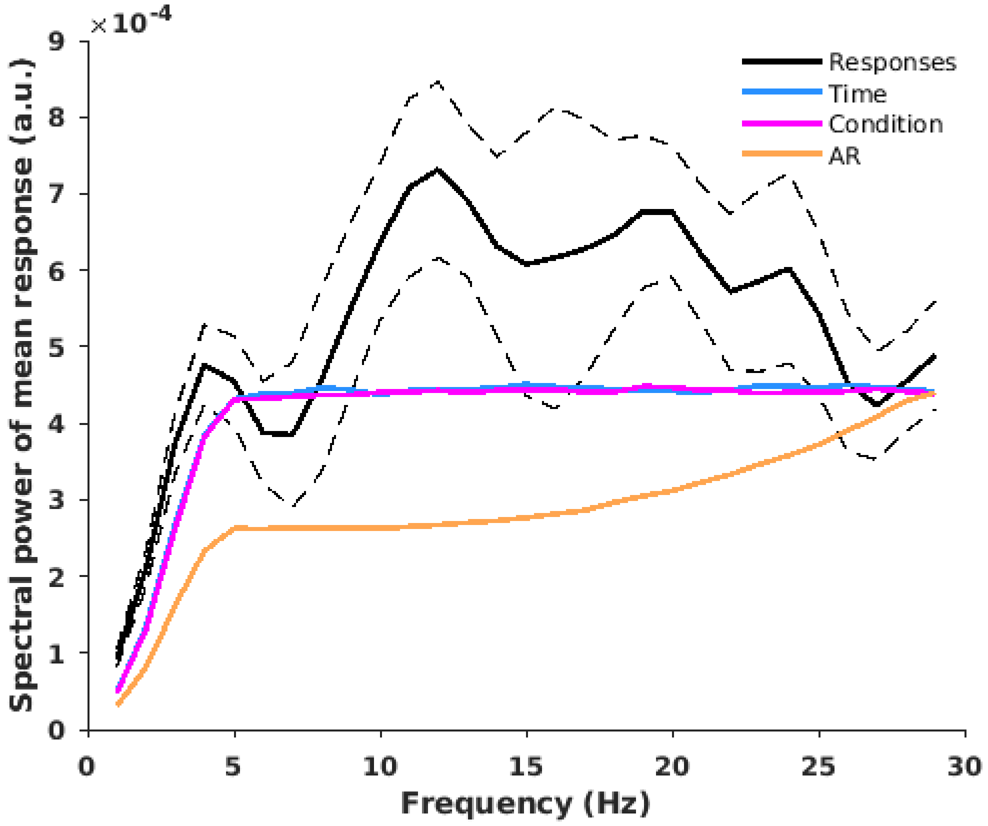

Supplementary Figure 1: Spectral power in comparison with surrogate datasets. On the x-axis is the spectral power as the mean response plotted against the frequency in hertz (y-axis). The mean response over all participants is the black solid line. The black dotted lines are the standard error of the mean. The colored lines are the mean surrogate data for each analysis. Blue shows the surrogate data for the time shuffled data. The pink one for the condition shuffled and the orange one for the AR shuffled data set.

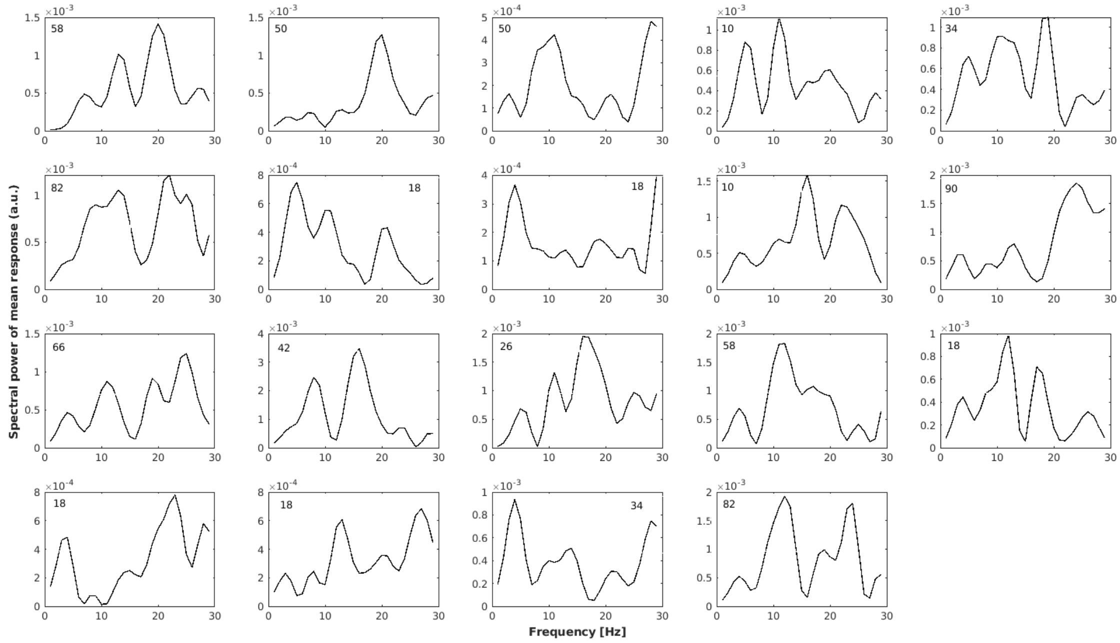

Supplementary Figure 2: Single subject plot for the behavioral spectral power. Each plot shows a single subject and the individual spectral power, plotted on the y-axis on the x-axis is the frequency in hertz.
